# ALKHB5-demethylated lncRNA SNHG15 promotes myeloma tumorigenicity by increasing chromatin accessibility and recruiting H3K36me3 modifier SETD2

**DOI:** 10.1152/ajpcell.00348.2023

**Published:** 2023-12-25

**Authors:** Lan Yao, Tingting Li, Yao Teng, Jingjing Guo, Hongyong Zhang, Linghui Xia, Qiuling Wu

**Affiliations:** Institute of Hematology, Union Hospital, Tongji Medical College, Huazhong University of Science and Technology, Wuhan, People’s Republic of China

**Keywords:** chromatin accessibility, long noncoding RNA, multiple myeloma, N6-methyladenosine, trimethylated histone H3 at lysine 36

## Abstract

Chromatin instability plays a crucial role in multiple myeloma (MM) relapse and progression, but its mechanism remains obscure. Here, we uncovered that m6A-demethylase ALKBH5 upregulated and stabilized long noncoding RNA (lncRNA) small nucleolar RNA host gene 15 (SNHG15), which was elevated in MM and positively correlated with unfavorable clinical prognosis factors. ALKBH5-SNHG15 axis participated in viability and migration/invasion of myeloma cell lines and MM-xenografted SCID/NOD mice. Mechanically, ALKBH5 promoted the expression of trimethylated histone H3 at lysine 36 (H3K36me3) methyltransferase SETD2 through lncRNA SNHG15-mediated protein stability. ALKBH5-SNHG15 axis increased chromatin accessibility and altered the H3K36me3 enrichment at the gene body, which is responsible for transcription elongation. Our study suggested a novel epigenetically interaction of *N*6-methyladenosine (m6A) methylation, lncRNA SNHG15, and histone SETD2/H3K36me3 modifications in myeloma progression, indicating that ALKBH5 and lncRNA SNHG15 could serve as potential novel therapeutic targets for MM treatment.

**NEW & NOTEWORTHY** To our knowledge, this study first demonstrated the prognostic significance and biological function of long noncoding RNA (lncRNA) small nucleolar RNA host gene 15 (SNHG15) in multiple myeloma (MM), and indicated a novel revelation on the effect of *N*6-methyladenosine (m6A)-regulated lncRNA on MM tumorigenicity. Moreover, the novel chromatin-regulatory mechanism of lncRNA by interacting with epigenetic modifiers including m6A demethylase ALKBH5 and H3K36me3 methyltransferase SETD2 in myeloma progression elucidated intricate mechanism of tumor pathogenesis.

## Graphical abstract

**Figure:**
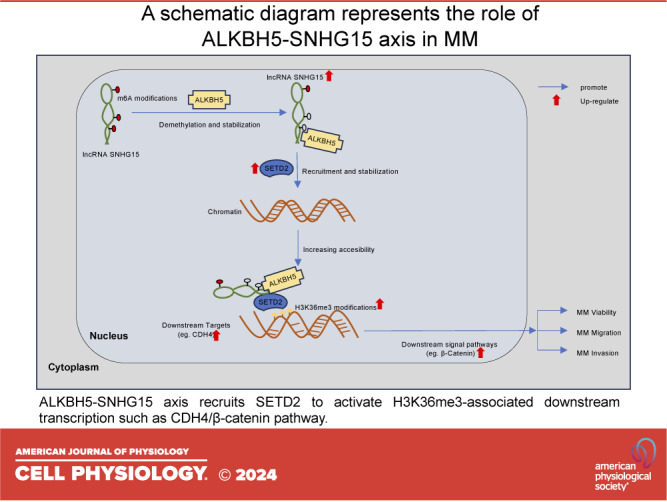

## INTRODUCTION

Multiple myeloma (MM) is the second most common hematological malignancy, manifested by uncontrolled proliferation of monoclonal plasma cells in the bone marrow (BM) (1). Although advanced therapeutic strategies such as immunomodulatory drugs, proteasome inhibitors, CD38 monoclonal antibodies, and chimeric antigens receptor-T cell therapy have greatly prolonged the overall survival of patients with MM, MM remains incurable, and almost all patients will eventually relapse. In-depth investigation is needed and expected to reveal the detailed mechanisms and improve patient prognosis. Chromatin instability plays a crucial role in MM relapse and progression, but its mechanism remains obscure.

RNA *N*6-methyladenosine (m6A), the most abundant internal RNA modification of eukaryotic RNA, is established by m6A methyltransferases such as METTL3, METTL14, and WTAP, and removed by demethylases FTO and ALKBH5. These m6A regulators posttranscriptionally manipulate target RNA metabolism and expression in diverse physiological and pathological processes, especially in tumorigenesis (2). In MM, accumulated evidence identified the decreased global RNA m6A level of MM cells than that of normal controls, suggesting the vital role of m6A demethylases in myelomagenesis (3–7). Our previous research has demonstrated that ALKBH5 promoted stem cell phenotype and myeloma tumorigenicity through demethylating the SAV1 mRNA of the HIPPO pathway, emphasizing the demethylation activity of ALKBH5 in MM (3). However, the detailed epigenetic mechanism involving ALKBH5 in MM is largely unknown and warrants more deep investigation.

Long noncoding RNAs (lncRNAs), as ncRNAs longer than 200 nucleotides, display copious roles in tumor initiation, growth, and metastasis via interacting with versatile DNA, RNA, and proteins (8, 9). In MM, Agirre and coworkers (10) performed paired-end ssRNA-seq of plasma cells (PCs) purified from the bone marrow (BM) of 38 patients with MM and revealed that lncRNAs accounted for 82% of MM transcriptome, whereas coding transcripts represented only 18%. Furthermore, coefficient of variation analysis showed that the heterogeneity of expression in MM samples was significantly higher for lncRNAs than for coding genes (10). Many well-studied cancer-related lncRNAs have been found dysregulated and executing regulatory or structural roles in MM. Recent findings have demonstrated that some m6A-modified lncRNAs could impact tumorigenesis through interacting and coregulating with m6A factors in leukemia and some solid malignancies, implying future research direction and potential clinical application. For instance, ALKBH5 stabilized lncRNA NEAT1 transcript via m6A demethylation and thus facilitated lncRNA NEAT1-mediated paraspeckle assembly, ultimately promoting glioblastoma multiforme progress through upregulating downstream CXCL8/IL8 pathway (11). However, whether RNA m6A methylation as the posttranscriptional epigenetic mechanism contributes to lncRNA dysregulations in MM has been rarely reported.

LncRNA SNHG15 has been reported to be overexpressed in various solid tumors such as pancreatic cancer, colorectal cancer, and glioblastoma, serving as a prospective biomarker for molecular diagnosis and therapeutics (12). It acted as a miR-4735-3p sponge to regulate hypoxia-inducible factor (HIF)-1a expression and promote tumorigenic properties and chemoresistance in cervical cancer (13). However, the role of SNHG15 in MM is still unclear. As the specific subcellular location of lncRNAs determines their function, whether lncRNA SNHG15 impacts myelomagenesis through its small nucleolar RNA (snoRNA) or the miRNA sponging function remains to be clarified.

In this study, we demonstrated the modulation of ALKBH5 on lncRNA SNHG15 and its role in MM pathogenesis and progression. We further identified the function of ALKBH5-SNHG15 axis on transcriptional control via chromatin accessibility regulation and histone modification, underlying the epigenetic network in MM.

## MATERIALS

### Ethical Statement and Primary MM Cells

This study was approved by the Clinical Research Committee of Wuhan Union Hospital, Huazhong University of Science and Technology, Wuhan, People’s Republic of China. Written informed consent was obtained from all patients. Forty-six patients with MM and 12 healthy volunteers who visited our hospital from April 2019 to January 2022 were enrolled in this study.

Mononuclear cells were freshly isolated from the BM of patients with MM (*n* = 46) and volunteers (*n* = 12), and CD138^+^ cells were selected with CD138 microbeads (5 μg/mL) (Cat. No. 130-051-301, Miltenyi) according to the manufacturer’s instructions.

### Cell Lines and Lentivirus Transfection

Four human multiple myeloma cell lines were purchased from the National Collection of Authenticated Cell Cultures. Cells were cultured in RPMI-8226 medium with 10% fetal bovine serum, 100 U/mL penicillin, and 100 mg/mL streptomycin at 37°C in an atmosphere of 5% CO_2_.

Lentivirus particles were constructed and transfected according to the manufacturer’s instructions by GeneChem (Shanghai, PR China). After 72 h transfection, MM cells were screened by 2 μg/mL of puromycin for 48 h. The shRNAs used are listed in Supplemental Material S1.

### RNA Subcellular Fractionation, Extraction, and Quantitative Reverse Transcription Polymerase Chain Reaction

Total cellular fractions were divided into cytoplasmic and nuclear fractions using an RNA subcellular isolation kit (Cat. No. 25501, Active Motif, Shanghai, PR China).

Total RNA was extracted from cells using TRIzol reagent (Invitrogen, CA). The extracted RNA was first used to digest the genomic DNA and the cDNA was obtained by reverse transcription with HiScript III RT SuperMix for qPCR (R323-01, Vazyme, PR China) for lncRNA and mRNA according to the manufacturer’s instructions. The real-time PCR analyses were performed using HiScript II One Step qRT-PCR SYBR Green Kit (Q221-01, Vazyme, PR China) by StepOnePlus Real-Time PCR System (Applied Biosystems, Foster City, CA). GAPDH was used as a loading control, and relative expression was calculated using the 2^−ΔΔCT^ method. Each sample was analyzed in triplicate. All specific primers are listed in Supplemental Material S1.

### Protein Extraction and Western Blot Analysis

Whole cell lysates were prepared using RIPA buffer containing protease inhibitors. After boiling, the supernatants were subjected to SDS-PAGE and transferred to PVDF membranes. After blocking with 5% nonfat milk, membranes were incubated with primary antibodies at 4°C overnight and detected by horseradish peroxidase (HRP)-conjugated secondary antibodies using enhanced chemiluminescence (Biosharp, PR China) by Bio Spectrum 600 Imaging System (UVP). All specific antibodies and their amount are listed in Supplemental Material S1.

### RNA Stability Assay with Actinomycin D

Cells (2 × 10^4^ cells/well) were plated in 12-well plates with actinomycin D (Act D) (Cat. No. HY-17559, MCE, Jinan, PR China) at a final concentration of 5 µg/mL. After 0-, 2-, 4-, and 6-h incubations, cells were collected, and RNA was isolated for quantitative reverse transcription polymerase chain reaction (RT-qPCR).

### Protein Stability Assay with Cycloheximide

Cells (8 × 10^5^ cells/well) were plated in six-well plates with cycloheximide (CHX) (Cat. No. HY-12320, MCE, Jinan, PR China) at a final concentration of 50 µg/mL. After 0- and 4-h incubations, cells were collected, and protein was isolated for Western blot.

### Demethylation Treatment with 3-Deazaadenosine

Cells (2 × 10^4^ cells/well) were plated in 12-well plates with 3-deazaadenosine (DAA) (Cat. No. HY-17559, MCE, Jinan, PR China) at a final concentration of 10 µg/mL for 48 h. Cells were then collected for RT-qPCR.

### Cell Viability, Apoptosis, and Transwell Assay

Cell viability was determined with the Cell Counting Kit 8 (Cat. No. A311-01/02, VAZYME, Nanjing, PR China). Cell apoptosis assay was determined according to the Annexin V-APC/7-AAD apoptosis kit (Cat. No. AP105, MULTI SCIENCE, Hangzhou, PR China), and data were analyzed by FlowJo software. Transwell assays were used to assess cell-migration/invasive ability with the Transwell system precoated without/with Matrigel (Cat. No. 356234, Corning, Shanghai, PR China).

### Methylated RNA Immunoprecipitation Sequencing

Methylated RNA immunoprecipitation sequencing (MeRIP-seq) was performed as previously reported with slight modifications (14). Briefly, 50 μg of total RNAs were polyadenylated RNA enriched, cut into 100–200 nt by adding 20 mM ZnCl_2_ at 95°C for 5–10 min, then incubated with m6A primary antibody (Cat. No. ab208577, Abcam, 1:100) and immunoprecipitated using Protein A Magnetic Beads (Cat. No. HY-K0203, MCE). RNA samples of both input and immunoprecipitation (IP) were prepared using TRIzol. The stranded RNA sequencing library was enriched, quantified, and finally sequenced on Novaseq 6000 sequencer (Illumina) with the PE150 model. More details on the study design are detailed in the Supplemental Material S1.

### Chromatin Immunoprecipitation-Sequencing

Chromatin immunoprecipitation-sequencing (CHIP-seq) was performed as previously reported with slight modifications (15). Briefly, cell samples were fixed in 1% formaldehyde for 10 min at room temperature and then added with 0.125 M glycine for 5 min to generate DNA-protein cross links. After being treated with cell and nucleus lysis buffer, the chromatin DNA fragments were collected by centrifuging at 2,000 *g* for 5 min and sonicated successively. The 10% lysis sonicated chromatin was stored as “input”, 80% was used in immunoprecipitation reactions with anti-H3K36me3 antibody (Cat. No. ab9050, Abcam, 1:100) as “IP”, and 10% was incubated with IgG as a negative control “IgG”, respectively. The DNA of input and IP was extracted by phenol-chloroform method, and the high-throughput DNA sequencing libraries were enriched, quantified, and finally sequenced on a Novaseq 6000 sequencer (Illumina) with PE150 model. More details on the study design are in the Supplemental Material S1.

### Gene-Specific RNA Binding Protein Immunoprecipitation-qPCR

RNA binding protein immunoprecipitation (RIP) was performed as previously described with slight modifications (16). Cleared by centrifugation at 14,000 rpm, the entire lysates from the supernatant were incubated with antibodies against m6A, and IgG (Cat. No. GB111738, Servicebio, 1:100) as control overnight at 4°C. Protein A beads were used to recover the RNA-protein complex. Following reverse cross linking with proteinase K at 50°C for 60 min, the protein-bound RNA was extracted using sodium acetate and ethanol. The relative abundance was examined by RT-qPCR.

### RNA Pulldown

Pulldown assay was performed as previously described (17). Briefly, cells were fixed with 4% paraformaldehyde, lysed, sonicated, and centrifugated. Supernatant (50 μL) was maintained as input, and the remaining was mixed with biotin-labeled antisense probes (designed and produced by Genecreate, Wuhan, PR China) or sense probes (designed and produced by Genecreate, Wuhan, PR China) and incubated with streptavidin C1 magnetic beads at 4°C overnight. Then, the beads were washed thoroughly with lysis buffer for at least three times. The RNA-protein binding mixture was boiled in an SDS buffer, and the proteins were detected by Western blot analysis.

### Co-Immunoprecipitation

Cells were lysed in co-immunoprecipitation (Co-IP) buffer supplemented with a protease inhibitor cocktail for 30 min on ice. Cell lysates were incubated with the indicated antibodies adsorbed to protein A microbeads for 4 h at 4°C before washing three times in Co-IP buffer and elution at 95°C for 10 min. Further protein-protein interaction was detected by Western blot analysis.

### DNase I-TUNEL Staining

DNase I-TUNEL experiment was performed according to a previous study (18). After fixed in 4% paraformaldehyde, cell slides were permeabilized by 0.5% Triton X-100 in PBS buffer for 15 min before digesting with 0.2 U/mL of DNase I (Cat. No. E-CK-A325, Elabscience). TUNEL assays kit (Cat. No. E-CK-A325, Elabscience) was subsequently used according to the manufacturer’s instructions. The nuclear area was defined according to DAPI DNA staining. Images were captured with an Olympus fluorescence microscope.

### Immunofluorescence

Cell slides were fixed and permeabilized. After blocking, slides were incubated with the primary and secondary antibodies, stained with DAPI (GDP1024, Servicebio), and finally imaged with a Nikon A1R SI confocal microscope.

### RNA Fluorescent In Situ Hybridization

RNA fluorescent in situ hybridization (FISH) was performed with a lncRNA SNHG15 specific probe (designed and produced by RIBOTM, Guangzhou) and RIBOTM FISH Kit (Cat. No. R11060.7). Briefly, cell slides were fixed with 4% paraformaldehyde at room temperature for 10 min, permeabilized with 0.5% Triton-X at 4°C room temperature for 5 min, and then incubated with lncRNA SNHG15 probe overnight at 37°C. After washing, the slides were stained with DAPI and imaged with a Nikon A1R SI confocal microscope.

### Animal Experiments

All animal experiments were approved by the Institutional Animal Care and Use Committee of Huazhong University of Science and Technology (No. 3569).

#### MM-xenografted model.

A total of 1 × 10^7^ cotransfected RPMI8226 MM cells, control RPMI8226 MM cells (shNC + LV-NC), ALKBH5-depleted RPMI8226 MM cells (shALKBH5 + LV-NC), or ALKBH5-depleted-SNHG15-overexpressed RPMI8226 MM cells (shALKBH5 + LV-SNHG15), were subcutaneously injected into the right flanks of the 4-wk-old male NOD/SCID mice (*n* = 6 for each group) to establish a human MM-xenografted model. Tumor growth was monitored every 3 days. The mice were euthanized after 4 wk, and tumors were measured (Tumor volume = π/6 × length × width^2^) and harvested.

#### Immunohistochemical staining.

Paraffin-embedded sections of tumor tissues from xenograft mice were incubated with primary antibodies at 4°C overnight. Subsequently, the sections were treated with polymer, developed with diaminobenzidine (DAB) chromogen, and counterstained with hematoxylin.

### Quantification and Statistical Analysis

In this study, all bioinformatic analysis was carried out using *R* software 4.1.3 (https://cran.r-project.org/bin/windows/base/). All differences were screened out according to the following criteria: FC (fold change) ≥ 1.5 and false discovery rate < 0.05.

Data were expressed as means ± SD, and analyses were performed by Prism GraphPad 9. The mean values were calculated on the basis of data obtained from at least three independent experiments conducted on separate days using freshly prepared reagents. The unpaired two-tailed Student’s *t* test was used to determine significant differences between the two groups, whereas one-way analysis of variance (ANOVA) followed by Tukey’s post hoc test assessed differences among multiple groups. Spearman correlation analysis was performed to determine the correlation between ALKBH5 mRNA and lncRNA SNHG15. *P* values of <0.05 were considered statistically significant.

## RESULTS

### ALKBH5 Stabilizes lncRNA SNHG15 in an m6A-Demethylated Way in MM

**Figure 1.**
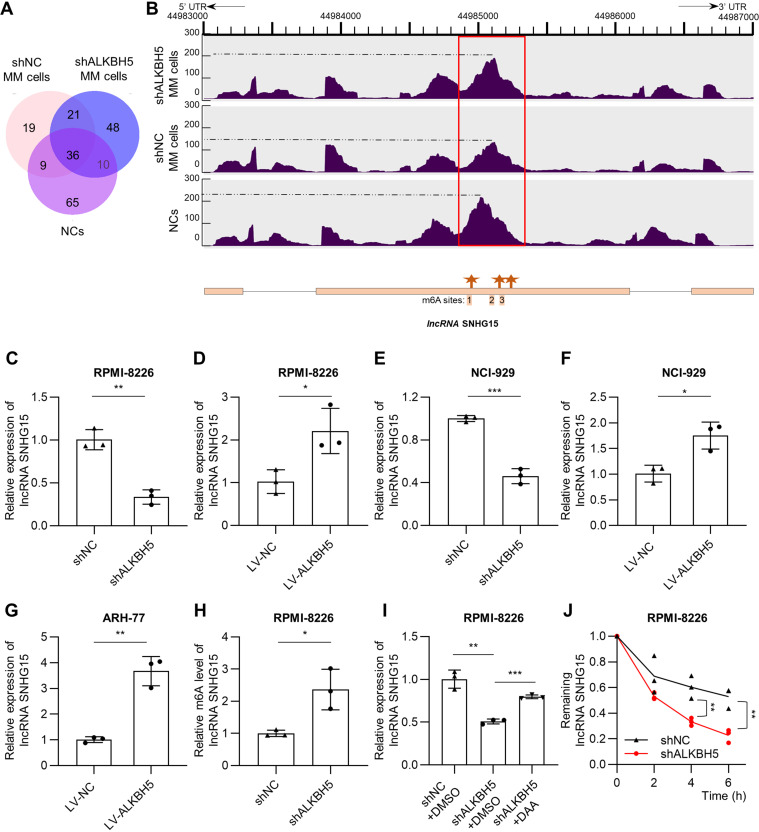
ALKBH5 demethylates and stabilizes long noncoding RNA (lncRNA) small nucleolar RNA host gene 15 (SNHG15) in multiple myeloma (MM). *A*: Venn diagram shows 10 lncRNAs with *N*6-methyladenosine (m6A) abundance both in ALKBH5-depleted RPMI8226 MM cells (shALKBH5) and bone marrow-derived CD138^+^ cells from healthy volunteers (normal controls, NCs), but no m6A abundance in control RPMI8226 MM cells (shNC) by methylated RNA immunoprecipitation sequencing (MeRIP)-sequencing. (2 independent biological replicates for each group). *B*: m6A abundance by MeRIP-seq analysis and the structure with potentially specific ALKBH5-demethylated m6A sites predicted by SRAMP of lncRNA SNHG15 in above MM cells. *C* and *D*: relative expression of lncRNA SNHG15 in shALKBH5 (*C*) and LV-ALKBH5 (*D*) RPMI8226 MM cells compared with shNC and LV-NC RPMI8226 MM cells, respectively, determined by quantitative reverse transcription polymerase chain reaction (RT-qPCR). *E* and *F*: relative expression of lncRNA SNHG15 in shALKBH5 (*E*) and LV-ALKBH5 (*F*) NCI-H929 MM cells compared with shNC and LV-NC NCI-H929 MM cells respectively determined by RT-qPCR. *G*: relative expression of lncRNA SNHG15 in LV-ALKBH5 ARH-77 MM cells compared with LV-NC ARH-77 MM cells determined by RT-qPCR. *H*: MeRIP-qPCR analysis of m6A levels of lncRNA SNHG15 in RPMI8226 MM cells after ALKBH5 knockdown. *I*: LncRNA SNHG15 levels in RPMI8226 cells with or without ALKBH5 depletion in response to demethylation treatment with 3-deazaadenosine (DAA) by RT-qPCR. *J*: the stability of lncRNA SNHG15 in RPMI8226 MM cells with or without ALKBH5 depletion was measured by RT-qPCR. Data were means ± SD of three independent experiments performed in triplicate. The unpaired two-tailed Student’s *t* test was used to determine significant differences between the two groups, whereas one-way analysis of variance (ANOVA) followed by Tukey’s post hoc test assessed differences among multiple groups. *P < 0.05, **P < 0.01, ***P < 0.001.

To interpret the function of ALKBH5 in MM extensively, we identified lncRNA small nucleolar RNA host gene 15 (SNHG15) as an ALKBH5-demethylated lncRNA by methylated RNA immunoprecipitation sequencing (MeRIP-seq). Briefly, through comparing the m6A abundance in lncRNAs among normal controls (NCs), shNC MM cells (using a control shRNA lentiviral vector, shNC), and ALKBH5-depleted RPMI8226 MM cells (using an shRNA lentiviral vector targeting ALKBH5, shALKBH5), specific peak analysis recognized 75 lncRNAs as NCs-specific lncRNAs, which showed m6A markers in NCs but no m6A markers in shNC MM cells. Using the same method, we identified 58 shALKBH5-specific lncRNAs compared with shNC MM cells. Since ALKBH5 is an m6A demethylase highly expressed in MM, 10 lncRNAs by the intersection of NCs-specific lncRNAs and shALKBH5-specific lncRNAs were recognized as potential ALKBH5-regulated lncRNAs (Fig. 1*A*, Supplemental Table S1). The knockdown efficiency of shALKBH5 is shown in Supplemental Fig. S1*A* (*P* < 0.01), which has been validated in our latest study (3). Notably, lncRNA SNHG15 had the most significant repressed-expression after ALKBH5 knockdown in RPMI8226 cells (*P* < 0.01, Supplemental Fig. S1*B*). As shown in Fig. 1*B*, compared with shALKBH5 MM cells and NCs cells, the m6A abundance of lncRNA SNHG15 in the red box area was lower in shNC MM cells, where three m6A sites potentially exist according to RMVar database (https://rmvar.renlab.org/) (19). Therefore, lncRNA SNHG15 was regarded as the downstream target of ALKBH5 for further investigation.

In RPMI8226 cells, SNHG15 level decreased with knockdown of ALKBH5 (Fig. 1*C*, Supplemental Fig. S1*A*), whereas SNHG15 level increased with overexpression of ALKBH5 (Fig. 1*D*, Supplemental Fig. S1*C*). Similar results were also observed in NCI-H929 and ARH-77 MM cells (*P* < 0.05, Fig. 1, *E–G*, Supplemental Fig. S1, *D*–*F*). MeRIP-qPCR determined that ALKBH5 knockdown increased the m6A methylation level of lncRNA SNHG15 in RPMI8226 cells compared with the control (*P* < 0.05, Fig. 1*H*). Furthermore, the decrease of lncRNA SNHG15 expression in ALKBH5-depleted RPMI8226 cells was attenuated by adding the global methylation inhibitor 3-deazaadenosine (DAA) (*P* < 0.01, Fig. 1*I*). After blocking new RNA synthesis with actinomycin D, lncRNA SNHG15 stability was markedly impaired after ALKBH5 depletion (*P* < 0.01, Fig. 1*J*). These results indicated that ALKBH5 upregulates and stabilizes lncRNA SNHG15 through its m6A demethylation activity.

### LncRNA SNHG15 Is Elevated in Patients with MM and MM Cell Lines

**Figure 2.**
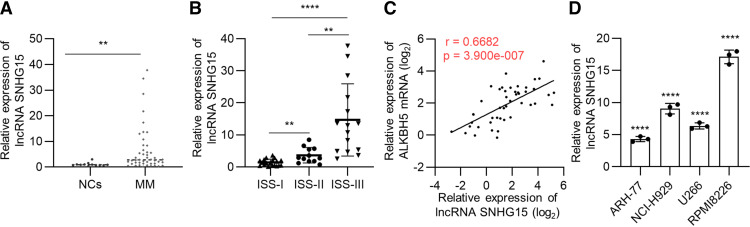
Long noncoding RNAs (LncRNA) small nucleolar RNA host gene 15 (SNHG15) is highly expressed in multiple myeloma (MM) and predicts poor prognosis. *A*: quantitative reverse transcription polymerase chain reaction (RT-qPCR) results of lncRNA SNHG15 expression in primary bone marrow-derived CD138^+^ cells from patients with MM (*n* = 46) compared with that from normal controls (NCs, *n* = 12). *B*: RT-qPCR results of lncRNA SNHG15 expression in primary MM cells from different disease stages. ISS I (*n* = 19), ISS II (*n* = 12), ISS III (*n* = 15). *C*: Spearman’s correlation analysis of lncRNA SNHG15 expression with ALKBH5 mRNA expression in primary MM cell samples. *D*: RT-qPCR results of lncRNA SNHG15 expression in four MM cell lines, compared with NCs. Data were means ± SD of three independent biological replicates performed in triplicate. The unpaired two-tailed Student’s *t* test was used to determine significant differences between the two groups, whereas one-way analysis of variance (ANOVA) followed by Tukey’s post hoc test assessed differences among multiple groups. **P < 0.01, ****P < 0.0001.

Then we compared the expression level of lncRNA SNHG15 between BM-derived CD138^+^ cells from 46 patients with MM and 12 NCs. The results showed that the expression of lncRNA SNHG15 in patients with MM was significantly higher than that in NCs (*P* < 0.01, Fig. 2*A*). Subgroup analysis found that lncRNA SNHG15 expression was positively correlated with the advanced MM stage (International Staging System stage) (*P* < 0.01, Fig. 2*B*), which indicates a poor prognosis. Moreover, correlation analysis validated the positive correlation between the expression level of SNHG15 and ALKBH5 (*r* = 0.6682, *P* = 0.00000039, Fig. 2*C*). Besides, SNHG15 was universally overexpressed in MM cell lines and most highly in RPMI8226 and NCI-H929 (*P* < 0.0001, Fig. 2*D*). Collectively, these findings suggest that elevated expression of lncRNA SNHG15 predicts advanced disease stage and poor clinical prognosis in MM.

### ALKBH5-SNHG15 Axis Promotes Myelomagenesis In Vitro

**Figure 3.**
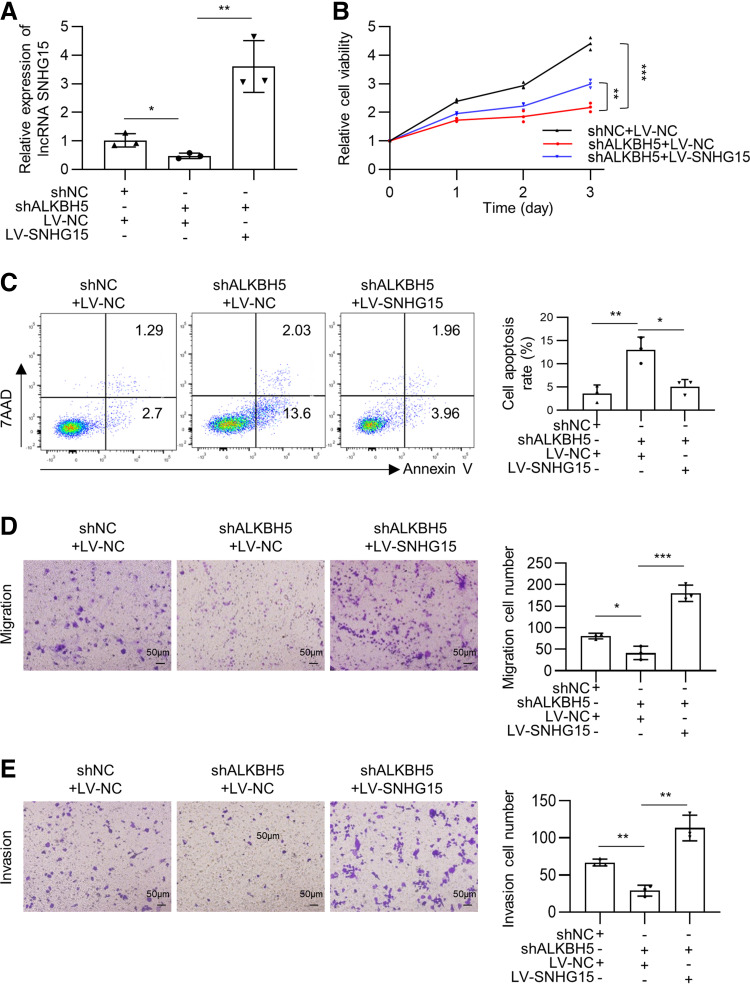
ALKBH5 promotes myelomagenesis through long noncoding RNA (lncRNA) small nucleolar RNA host gene 15 (SNHG15) in RPMI8226 MM cells. *A*: relative expression of lncRNA SNHG15 in RPMI8226 MM cells cotransfected with shALKBH5 and LV-SNHG15 was determined by quantitative reverse transcription polymerase chain reaction (RT-qPCR). *B*: the viability of RPMI8226 cells cotransfected with shALKBH5 and LV-SNHG15 was examined using CCK8 assay. *C*: the representative images and proportion of apoptotic cells in cotransfected RPMI8226 cells were measured using flow cytometry analysis. *D* and *E*: the altered migration (*D*) and invasion (*E*) ability of cotransfected RPMI8226 cells were measured using Transwell assay, Scale bar = 100 μm. Data were means ± SD of three independent biological replicates performed in triplicate. One-way ANOVA with Tukey post hoc test. *P < 0.05, **P < 0.01, ***P < 0.001.

Ectopic expression of SNHG15 in ALKBH5-depleted RPMI8226 cells were used to determine whether lncRNA SNHG15 is involved in ALKBH5-mediated myelomagenesis (*P* < 0.05, Fig. 3*A*). CCK8 assay showed that the restoration of lncRNA SNHG15 expression partially rescued the antiviability effect of ALKBH5 knockdown in RPMI8226 cells (*P* < 0.01, Fig. 3*B*). Flow cytometry experiments manifested that the increased apoptosis induced by ALKBH5 knockdown was alleviated by SNHG15 overexpression (*P* < 0.05, Fig. 3*C*). Transwell assay showed that diminished migration/invasion ability attributed to ALKBH5 depletion was offset by lncRNA SNHG15 overexpression (*P* < 0.05, Fig. 3, *D* and *E*). Reversely, lncRNA SNHG15 depletion in ALKBH5-overexpressed NCI-H929 cells rescued the effects of ALKBH5 overexpression on MM viability and migration/invasion (*P* < 0.05, Supplemental Fig. S2, *A*, *B*, *D*, and *E*). However, although there was a statistical difference, the apoptosis rate altered slightly after SNHG15 depletion on ALKBH5-overexpressed NCI-H929 cells, which might be attributed to the low baseline apoptosis proportion of myeloma cells (Supplemental Fig. S2*C*). These results suggest that lncRNA SNHG15 participates in ALKBH5-mediated cell viability and migration/invasion in MM. Since the antiapoptosis effect of SNHG15-overexpression on ALKBH5-depleted MM cells was not thoroughly rescued (Fig. 3*C*), it seems that the antiapoptosis of ALKBH5 might be partially mediated through SNHG15.

Moreover, four independent shRNAs were designed to target SNHG15, and the shRNA no. 1 showed the strongest silencing efficiency (nearly 50%) in both two MM cell lines (*P* < 0.01, Supplemental Fig. S1*G*). After ablating SNHG15 expression, a series of loss-of-function experiments revealed that lncRNA SNHG15 depletion inhibited cell viability, slightly induced cell apoptosis, and impaired cell migration/invasion in MM cells (*P* < 0.01, Supplemental Fig. S3, *A*–*H*). Therefore, ALKBH5-demethylated lncRNA SNHG15 is clinically and functionally oncogenic in MM, emerging as a new therapeutic target.

### ALKBH5-SNHG15 Axis Opens Chromatin and Activates Transcription through Stabilizing SETD2

**Figure 4.**
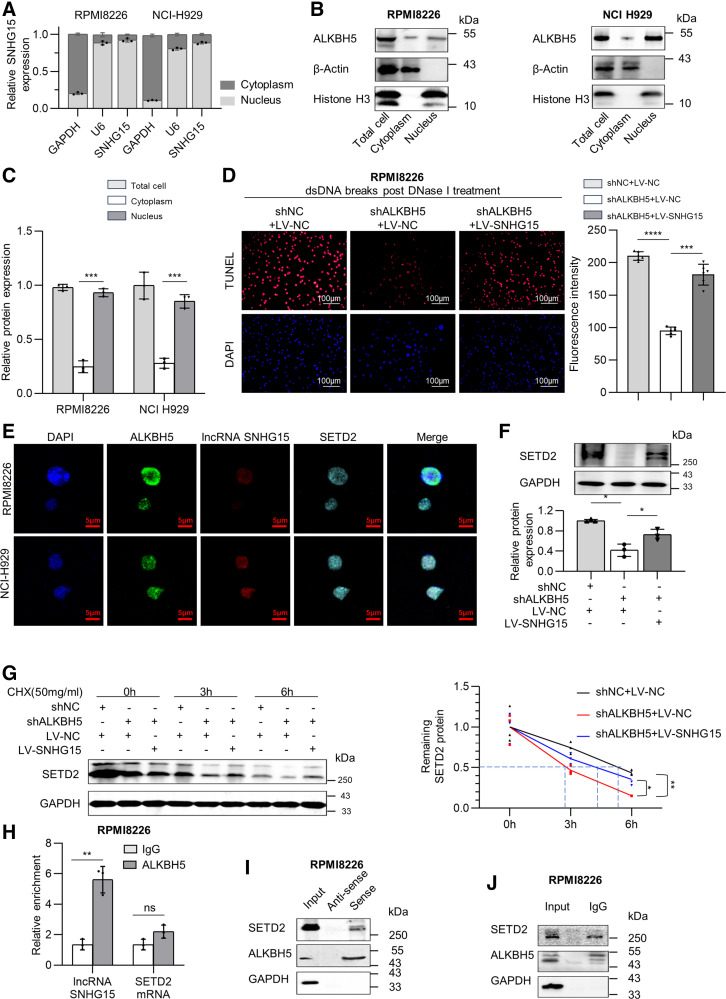
Nuclear-located ALKBH5 interacts with and stabilizes SETD2 protein to open chromatin and activate transcription via *N*6-methyladenosine (m6A)-demethylated long noncoding RNAs (lncRNA) small nucleolar RNA host gene 15 (SNHG15). *A*: quantitative reverse transcription polymerase chain reaction (RT-qPCR) examined the cytoplasmic and nuclear distribution of lncRNA SNHG15 in RPMI8226 and NCI-H929 cells. GAPDH and U6 were applied as positive controls in the cytoplasm and nucleus. *B* and *C*: the cytoplasmic and nuclear distribution of ALKBH5 protein was examined by Western blot in RPMI8226 and NCI-H929 cells. β-Actin and Histone H3 were applied as positive controls in the cytoplasm and nucleus. *D*: analysis of chromatin accessibility in RPMI8226 cells cotransfected with shALKBH5 and LV-SNHG15 was performed by DNase I-treated TUNEL assay. Scale bar = 100 μm. *E*: cellular localization detection of lncRNA SNHG15, ALKBH5, and SETD2 in RPMI8226 and NCI-H929 cells by RNA fluorescent in situ hybridization (FISH) and immunofluorescence (IF) costaining. Scale bar = 5 μm. *F*: Western blot results of SETD2 expression in RPMI8226 cells cotransfected with shALKBH5 and LV-SNHG15. *G*: Western blot results of SETD2 expression in cotransfected RPMI8226 with cycloheximide (CHX) treatment. *H*: anti-ALKBH5 RNA immunoprecipitation (RIP) analysis of ALKBH5-binding transcripts in RPMI8226 cells. Enrichment of lncRNA SNHG15 and SETD2 mRNA was measured by RT-qPCR and normalized to input. *I*: RNA pulldown assay of lncRNA SNHG15-associated proteins in RMPI8226 cells using biotinylated lncRNA SNHG15 probes or antisense probes. ALKBH5, SETD2 was detected by Western blot. *J*: coimmunoprecipitation analysis of ALKBH5-binding proteins in RPMI8226 cells using an anti-ALKBH5 antibody. SETD2 was detected by Western blot. Data were means ± SD of three independent biological replicates performed in triplicate. The unpaired two-tailed Student’s *t* test was used to determine significant differences between the two groups, whereas one-way analysis of variance (ANOVA) followed by Tukey’s post hoc test assessed differences among multiple groups. *P < 0.05, **P < 0.01, ***P < 0.001, ****P < 0.0001.

Since RNA and protein subcellular location analysis revealed that both ALKBH5 and lncRNA SNHG15 are mainly located in the nucleus of RPMI8226 and NCI-H929 cells (Fig. 4, *A–C*), suggesting the nuclear-regulatory function of ALKBH5-lncRNA SNHG15 axis in MM. We then performed DNase I-TUNEL assay and observed a notable decrease in chromatin accessibility after ALKBH5 depletion in RPMI8226 MM cells, which was remedied by lncRNA SNHG15 overexpression (Fig. 4*D*), and opposite tendencies were found in NCI-H929 cells cotransfected with LV-ALKBH5 and shSNHG15 as aforementioned (Supplemental Fig. S4*A*). Therefore, we speculate that ALKBH5-SNHG15 axis activates gene transcription via increasing chromatin accessibility.

m6A methylation of RNAs has been reported to regulate transcription via opening chromatin (20). Recently, lncRNAs have been reported to epigenetically modulate gene transcription through interacting with chromatin modifiers and thus altering histone modifications (21–23), we then asked whether there is a certain chromatin modifier interacted with lncRNA SNHG15 or ALKBH5 to carry out the chromatin-regulatory function. The trimethylated histone H3 at lysine 36 (H3K36me3) methyltransferase SETD2 is prevalently mutated in many solid cancers and leukemia, and it is thought to modulate genomic instability and gene body-associated transcriptional elongation via catalyzing H3K36me3 (23–26). We found an abnormal increase of SETD2 in MM primary cells and MM cell lines (Supplemental Fig. S4*B*). Interestingly, immunofluorescence and fluorescence in situ hybridization assays demonstrated that lncRNA SNHG15, SETD2, and ALKBH5 were all mainly localized in the nucleus (Fig. 4*E*, Supplemental S4*C*). Knockdown of ALKBH5 or lncRNA SNHG15 in MM RPMI8226 cells markedly decreased SETD2 expression, respectively (Supplemental Fig. S4, *D* and *E*). LncRNA SNHG15 overexpression rescued the reduction of SETD2 expression by ALKBH5 depletion in PRMI8226 MM cells (Fig. 4*F*). Contrasting results were observed in cotransfected NCI-H929 cells (Supplemental Fig. S4*F*). It has been reported that lncRNA SNHG15 could interact with and stabilize chromatin-associated transcription factor (27), and therefore we speculated that ALKHB5 regulated SETD2 through lncRNA SNHG15-mediated stabilization. Per our speculation, cycloheximide (CHX) treatment revealed that ALKBH5-depletion decreased the half-life of SETD2 degradation compared with the control cells, which was increased by lncRNA SNHG15 rescue (Fig. 4*G*). Therefore, we confirm that ALKBH5-SNHG15 axis mediated SETD2 protein stability.

RNA binding protein immunoprecipitation (RIP)-qPCR assay using anti-ALKBH5 antibody indicated that ALKBH5 was able to enrich lncRNA SNHG15, but not SETD2 mRNA (Fig. 4*H*), suggesting that ALKBH5 modulated SETD2 in an m6A-independent way. Pulldown assay indicated the binding of lncRNA SNHG15 with both ALKBH5 and SETD2 (Fig. 4*I*). In addition, we carried out coimmunoprecipitation with anti-ALKBH5 antibody, which indicated the binding of ALKBH5 with SETD2 (Fig. 4*J*). All these results demonstrated that ALKBH5-SNHG15 axis recruited and combined H3K36me3 methyltransferase SETD2 to execute the chromatin-regulatory role. Further investigation is needed to confirm if the interaction is direct and to reveal the detailed mechanisms.

### ALKBH5-lncRNA SNHG15 Axis Promotes Myelomagenesis through Activating H3K36me3-Associated Transcription

**Figure 5.**
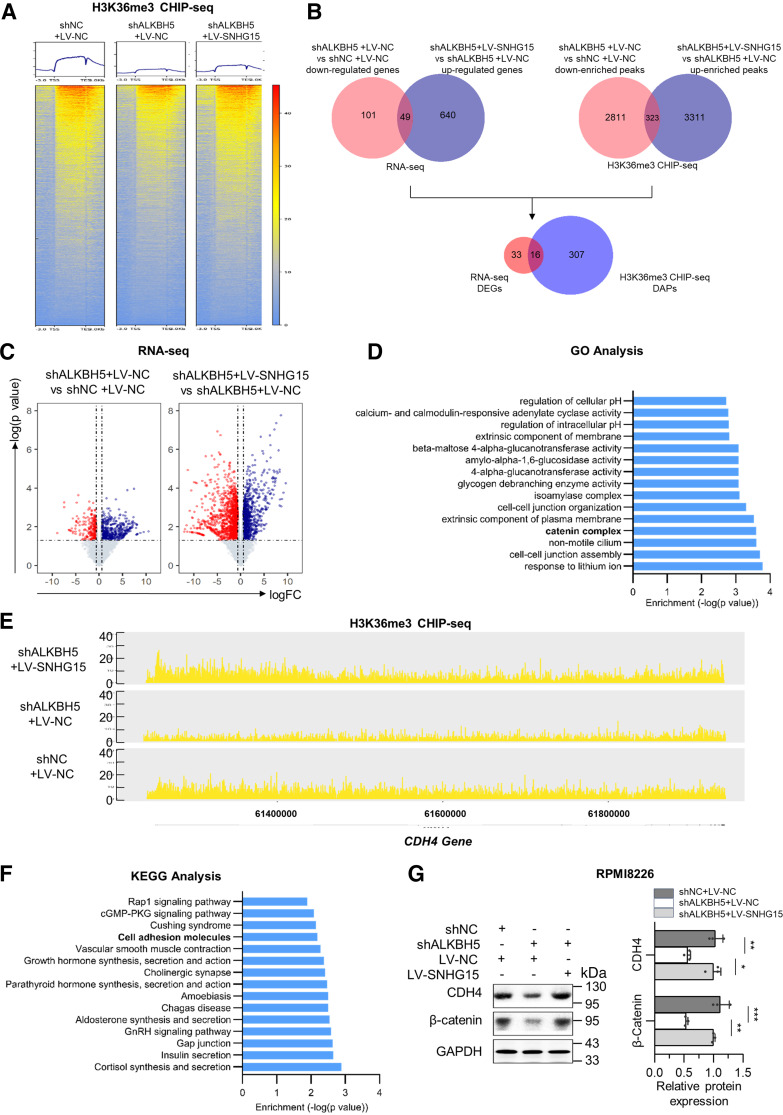
ALKBH5-long noncoding RNA (lncRNA) small nucleolar RNA host gene 15 (SNHG15) axis promotes myelomagenesis through the H3K36me3-activated CDH4/β-catenin pathway. *A*: heatmap of H3K36me signal from −3 kb of the TSS to +3 kb of the TES of the global genome in RMPI8226 MM cells cotransfected with shALKBH5 and LV-SNHG15. *B*: Venn diagrams identified 49 differentially expressed genes (DEGs) with over 1.5-fold expression change by RNA-Seq and 323 differentially accessible peaks (DAPs) with over 1.5-fold expression change by H3K36me3 chromatin immunoprecipitation (CHIP)-seq as the target genes of ALKBH5-SNHG15-SETD-targeted genes (≥2 independent biological replicates for each group). *C*: volcano plots showed the DEGs in shALKBH5+LV-NC RPMI8226 MM cells compared with control shNC + LV-NC RPMI8226 cells and in shALKBH5 + LV-SNHG15 RPMI8226 cells compared with shALKBH5 RPMI8226 cells. *D* and *F*: gene ontology (GO) and Kyoto Encyclopedia of Genes and Genomes (KEGG) analysis of 16 ALKBH5-SNHG15-SETD-targeted genes. *E*: CHIP-seq tracts for H3K36me3 at CDH4 gene locus in control RPMI8226 cells, ALKBH5-depleted RPMI8226 MM cells, and ALKBH5-depleted-SNHG15-overexpressed RPMI8226 cells. *G*: the Western blot results of CDH4 and β-catenin expression in cotransfected RPMI8226. Data were means ± SD of three independent biological replicates performed in triplicate. One-way ANOVA with Tukey post hoc test. *P < 0.05, **P < 0.01, ***P < 0.001.

SETD2 is the only human methyltransferase responsible for H3K36me3 modifications. In MM, H3K36me3 is supposed to be a transcriptionally active chromatin marker involved in the epigenetic regulation of gene expressions and chromatin instability (28). To further explore the effect of ALKBH5-lncRNA SNHG15 axis in chromatin regulation, H3K36me3 chromatin immunoprecipitation sequencing (CHIP-seq) was performed on control RPMI8226 MM cells (shNC + LV-NC), ALKBH5-depleted RPMI8226 MM cells (shALKBH5 + LV-NC), and ALKBH5-depleted-SNHG15-overexpressed RPMI8226 MM cells (shALKBH5 + LV-SNHG15) as aforementioned. After mapping the H3K36me3 peaks to normalizing input, we identified 42,634 H3K36me3 peaks from 8,422 genes in control MM cells, 37,219 H3K36me3 peaks from 8,073 genes in ALKBH5-depleted MM cells, and 43,045 H3K36me3 peaks from 8,653 genes in ALKBH5-depleted-SNHG15-overexpressed MM cells, confirming that ALKBH5-SNHG15 axis activated global transcription via H3K36me3 modifications. Distribution analysis revealed that global H3K36me3 levels were generally less enriched at the gene body in ALKBH5-downregulated MM cells than in control MM cells, which was re-enriched by lncRNA SNHG15 rescue (Fig. 5*A*). Taken together, we suggest that ALKBH5-SNHG15 axis opens chromatin and globally activates H3K36me3-associated transcription elongation in the gene body in MM.

To understand the regulatory role of ALKBH5-SNHG15 axis in comprehensive gene expression, we used RNA-Seq and integrated it with H3K36me3 CHIP-seq (The detailed workflow is depicted in Fig. 5*B* and Supplemental S4*H*). Briefly, we identified 150 downregulated differentially expressed genes (DEGs) with at least 1.5-fold after ALKBH5 knockdown (down-DEGs after ALKBH5 depletion), whereas 689 genes were upregulated by at least 1.5-fold after lncRNA SNHG15 rescue (up-DEGs after SNHG15 rescue) (Fig. 5*C*). By intersecting down-DEGs after ALKBH5 depletion and up-DEGs after SNHG15 rescue, 49 genes were defined as RNA-Seq DEGs. Considering that H3K36me3 is an active-transcriptional mark, we identified 323 differentially accessible peaks (DAPs) in H3K36me3 CHIP-seq using the same method. Consequently, by intersecting CHIP-seq with RNA-Seq, 16 genes were intersected and defined as target genes (Supplemental Table S2). Among them, the expression and abundance of ADCY1, WBSCR17, and CDH4 were altered the most. Further gene ontology (GO) and Kyoto Encyclopedia of Genes and Genomes (KEGG) analysis based on these 16 target genes was performed by clusterProfiler 4.0 R package (29). The results indicated their relationship with cell adhesion, junction, and catenin complex (Fig. 5, *D* and *F*, Supplemental Tables S3 and S4), which were all associated with CDH4, a cadherin family member modulating tumor proliferation and migration through the β-catenin pathway as a downstream target. After integrating the results of DEGs and Enrichment analysis, we chose CDH4 (Cadherin 4) for validation. By CHIP-analysis, H3K36me3 enrichment on the CDH4 gene locus was consistently decreased a half after ALKBH5 knockdown (the ratio of peak abundance: 0.5), which was remedied by lncRNA SNHG15 overexpression (the ratio of peak abundance: 2) (Fig. 5*E*). In addition, we observed that the decrease of CDH4 and β-catenin expression after ALKBH5-depletion were restored by SNHG15 overexpression in RPMI8226 cells (Fig. 5*G*), whereas the increase of CDH4 and β-catenin expression after ALKBH5-overexpression were attenuated by SNHG15 depletion in NCI-H929 cells (Supplemental Fig. S4*G*). In conclusion, ALKBH5-SNHG15 axis recruits SETD2 to activate H3K36me3-associated downstream transcription such as CDH4/β-catenin pathway.

### ALKBH5-lncRNA SNHG15 Axis Promotes Myeloma Tumorigenicity In Vivo

**Figure 6.**
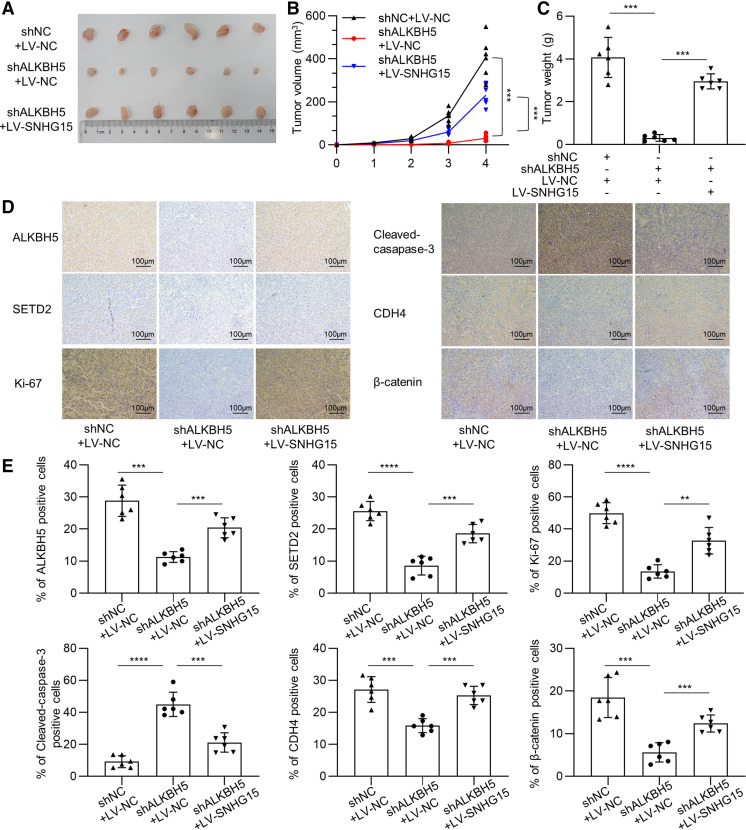
ALKBH5-long noncoding RNAs (lncRNA) small nucleolar RNA host gene 15 (SNHG15) axis promotes multiple myeloma (MM) tumorigenicity in vivo. *A*: tumor volume of NOD/SCID male mice xenografted with different groups of transfected RPMI8226 cells (*n* = 6). *B*: tumor growth curves of NOD/SCID mice after transfected RPMI8226 cells injection (*n* = 6). *C*: the weight of tumors at the end of treatment (*n* = 6). *D*: representative immunohistochemical staining of ALKBH5, SETD2, Ki-67, cleaved caspase-3, CDH4, and β-catenin in tumor xenografts. Scale bar = 100 μm. Data were means ± SD performed in six independent experiments. *E*: quantitation of immunohistochemical analysis of ALKBH5, SETD2, Ki-67, Cleaved-Caspase-3, and CDH4, β-catenin. One-way ANOVA with Tukey post hoc test. **P < 0.01, ***P < 0.001, ****P < 0.0001.

We then examined the effect of the ALKBH5-lncRNA SNHG15 axis on MM tumorigenicity by establishing MM-xenografted SCID/NOD mice. Mice were subcutaneously injected ion into mice with control RPMI8226 cells (shNC + LV-NC), ALKBH5-depleted RPMI8226 MM cells (shALKBH5 + LV-NC), and ALKBH5-depleted-SNHG15-overexpressed RPMI8226 cells (shALKBH5 + LV-SNHG15) as described in materials. Compared with the control group, mice injected with ALKBH5-depleted MM cells displayed slower tumor growth with decreased tumor burden while this inhibition was effectively rescued by lncRNA SNHG15 overexpression (*n* = 6, Fig. 6, *A–C*). Immunohistochemical analysis (IHC) was then used and displayed decreased expression of ALKBH5 and SETD2/H3K36me3 in ALKBH5-depleted mice, which were restored by lncRNA SNHG15 overexpression (Fig. 6, *D* and *E*). In addition, decreased staining of *K*_i_-67, CDH4, and β-catenin, and increased staining of cleaved caspase-3 were observed after ALKBH5 depletion, which was abrogated by lncRNA SNHG15 overexpression, indicating that ALKBH5-lncRNA SNHG15 axis modulates in vivo MM biological function via modulating SETD2/H3K36me3 and thus CDH4/β-catenin.

## DISCUSSION

In this study, we found that ALKBH5 upregulated and stabilized lncRNA SNHG15 in an m6A-demethylated way. ALKHB5-demethylated lncRNA SNHG15 was elevated in BM-derived CD138^+^ cells from patients with MM and MM cell lines, predicting poor prognosis. ALKHB5-demethylated lncRNA SNHG15 participated in MM cell viability, and migration/invasion in myeloma cell lines and MM-xenografted SCID/NOD mice. Further investigations revealed that protein expression promotion of H3K36me3 methyltransferase SETD2 by ALKBH5-SNHG15-mediated protein stability led to chromatin opening and transcription activation. Costaining analysis depicted the nucleus localization of ALKBH5, lncRNA SNHG15, and SETD2. Mechanism analysis revealed the binding of lncRNA SNHG15 with ALKBH5 and SETD2, implying they might form a chromatin-regulatory unit. H3K36me3 CHIP analysis showed that ALKBH5-SNHG15 axis altered the H3K36me3 enrichments at the gene body, which is responsible for transcription elongation. Our study suggested a novel epigenetic interaction of m6A methylation, lncRNA SNHG15, and histone SETD2/H3K36me3 modifications in myeloma progression.

LncRNA SNHG15 was highly expressed in MM and positively related to the advanced MM stage in patients with MM. Besides, to our knowledge, for the first time, a clinically positive correlation was found between the expression levels of ALKBH5 and lncRNA SNHG15, indicating that m6A-demethylated lncRNA SNHG15 may be a biomarker of poor prognosis in patients with MM. Both in vitro and in vivo studies revealed that ALKBH5-demethylated lncRNA SNHG15 regulates malignant phenotypes of MM, including viability and migration/invasion of MM cells, implying it is a novel and critical myeloma-promoting molecule and might be a potential therapeutic target of MM.

Besides, although we identified SNHG15 as a downstream target of ALKBH5 in MM, the expression level of ALKBH5 was restored after forced overexpression of SNHG15 (Fig. 6*D*). This indicated that ALKBH5 may act synergistically with SNHG15 like a positive feedback loop in both basal expression and biological function to promote MM tumorigenicity. The underlying mechanism leading to this interesting phenomenon still needs to be further revealed by subsequent studies.

H3K36me3, an actively transcribed histone mark, is related to transcriptional elongation of the gene body. To date, lncRNAs interact with various chromatin modifiers and serve as modular scaffolds of nearly all well-studied histone modifications (30, 31), including H3K27ac, H3K9ac, H3K27me3, H3K4me3, H4K20me3, and H3K9me3. However, few of them have been found mediating SETD2/H3K36me3-associated chromatin modulation. lncRNA MALAT1 has been reported to form a lncRNA-protein complex with SETD2 and other cofactor to modulate transforming growth factor (TGF)-β/Smad signaling in hepatic cells but have little effect on histone H3K36 methyltransferase activity, although they colocalized to the nuclear speckle (32). Our study found that ALKBH5-demethylated lncRNA SNHG15 interacted with and stabilized H3K36me3 methyltransferase SETD2, suggesting ALKBH5-demethylated lncRNA SNHG15 as a novel chromosome-associated regulatory RNA (carRNA) involved in H3K36me3 modifications. However, the role of SETD2 in myeloma progress remains elusive. Further investigation is needed to confirm if the interaction is direct and to reveal the detailed mechanisms.

In the past decade, research about the cofunctions of epigenetic mechanisms on chromatin regulation was mainly focused on the interaction between DNA methylation and histone modifications since it is widely acknowledged that DNA methylation cooperates with suppressing histone modifications or antagonizes activating-histone modifications to modulate chromatin accessibility and target gene transcription (33, 34). H3K36me3 was reported to bind METTL14 directly and facilitate m6A deposition on nascent RNAs to regulate gene expression cotranscriptionally (22, 35, 36). Recently, RNA m6A has been found to regulate chromatin (18). METTL3 localizes to intracisternal A particle (IAP)-type family of the endogenous retroviruses in mice embryonic stem cell via interaction with histone H3K9me3 methyltransferase SETDB1 and its cofactor (TRIM28). Catalyzing RNA m6A modification is essential for the integrity of IAP heterochromatin (22). In our study, RIP-qPCR, co-immunoprecipitation, RNA-pulldown, and costaining analysis validated the interaction of ALKBH5, lncRNA SNHG15, and SETD2, raising the possibility that they may constitute a triple complex and colocalize to specific chromatin regions, increasing chromatin accessibility and activating target gene transcription associated with MM tumorigenicity. Considering the short-lived characteristic of lncRNAs and our results that ALKBH5 stabilizes SETD2 through m6A-demethylating lncRNA SNHG15 instead of directly demethylating SETD2 mRNA (Fig. 4*H*), we postulated that ALKBH5 might act as a stabilizer of lncRNA SNHG15 to strengthen its interaction with SETD2. On the other hand, lncRNA SNHG15 may serve as a scaffold to sustain the codeposition and enhance the interaction of ALKBH5 and SETD2. More importantly, ALKBH5 might anchor steady to a particular target gene where SETD2 installs the H3K36me3 marks and finally promotes the target gene expression. Further study should focus on whether they form a steady tri-complex, and a relative truncation test is needed to investigate the detailed binding motif and domain in this unit. Conclusively, the interactive cofunctions of different epigenetic modifications alter chromatin structure and regulate gene expression, making the tumor pathogenesis mechanism a more complex network that deserves our attention.

## DATA AVAILABILITY

Data will be made available upon reasonable request.

## SUPPLEMENTAL DATA

10.6084/m9.figshare.24716301 Supplemental Material S1, Supplemental Tables S1–S4, and Supplemental Figs. S1–S4: https://doi.org/10.6084/m9.figshare.24716301.

## GRANTS

This work was totally supported by two grants from the National Natural Sciences Foundation of China under Grant Nos. 81570193 and 81770219 (to Q. Wu).

## DISCLOSURES

No conflicts of interest, financial or otherwise, are declared by the authors.

## AUTHOR CONTRIBUTIONS

L.Y., H.Z., L.X., and Q.W. conceived and designed research; L.Y., T.L., Y.T., J.G., and H.Z. performed experiments; L.Y., T.L., Y.T., J.G., H.Z., and Q.W. analyzed data; L.Y., T.L., Y.T., J.G., and H.Z. interpreted results of experiments; L.Y. prepared figures; L.Y. drafted manuscript; H.Z., L.X., and Q.W. edited and revised manuscript; H.Z., L.X., and Q.W. approved final version of manuscript.
